# Universal health coverage: Manifest destiny or Sisyphean pursuit?

**DOI:** 10.7189/jogh.11.01006

**Published:** 2021-08-28

**Authors:** Stephen Lewarne

**Affiliations:** Deloitte Consulting, Arlington, Virginia, USA

This project began with a debate and discussion between me and Professor Allyala Nandakumar (Nanda) in the Fall of 2018. Both of us were deeply involved in responding to United States Agency for International Development (USAID) and other donor programs in global health, public financial management (PFM), domestic resource mobilization (DRM), and private sector finance. My perspective was that of a USAID implementing partner, while Nanda’s as the Chief Economist for the Global Health Bureau at USAID. During that time, work being done by the World Bank (Dr Olusoji Adeyi and his colleagues) and USAID (Nanda and his colleagues) showed that even with rapid economic growth, few countries in Africa would be able to publicly fund an essential package of health benefits. Each of our previous work on the subject was calling for a more progressive and strategic approach to universal health coverage (UHC).

As we worked from both sides of various donor solutions, we asked critical questions of each other: “How effective have donors really been in meeting the Sustainable Development Goals (SDG) of UHC, and the broader health care needs of low- and middle-income country (LMIC) economies?;” “How sustainable are the solutions currently being proposed, especially with regard to universal health coverage?;” “Have we adequately addressed the full range of financing options available to even the poorest governments in meeting UHC objectives?” And finally, “Have we had an honest debate about the appropriate mix of the public and private provision of health care services necessary to meet the UHC objectives of the SDGs and stated by the United Nations (UN), and more practically those required in all their various forms, by the poorest countries of the world?”

The last question regarding the appropriate (optimal?) mix of public and private service provision focused our discussion around the political problems surrounding UHC and the financial solutions required. We both recognized that global health, even as the majority spending portion of USAID and other major donors, could not operate independent of PFM, DRM, nor private sector finance and development. If the UN and SDG UHC goals were to have any hope of “sustainability” (the hallmark criterion of most development agencies and certainly of USAID), the global health bureaus of the donor agencies would have to push global health programs to include a substantial dose of DRM, PFM, and private sector finance solutions.

Another critical part of Nanda’s and my debate was the recent push to define health care as a “right” particularly in some LMIC economies’ laws and even their Constitutions. The practicality of including a “service” (which health care is–in any form) as a fundamental “right” in the foundational legal documents of a country (eg, Ukraine) is dubious at best. Services must be paid for, and when they cannot, they cease to exist. Thus, countries that include such “rights” as health care in their fundamental laws are bound to run into constitutional crisis on a regular basis, as they will frequently (habitually?) lack the funding to service that “right.”

However, we felt this meta-debate on health care as a “right” clouded a more important issue. While one can argue the inclusion of a service (such as UHC) as a right, it does not take away from the fact that most LMICs see health care as critically important. And while we may wish to point out the constitutional pitfalls of including UHC in their foundational laws – that does not in any way reduce its political importance and thus the legitimacy of the government responsible for the provision of the service. The “rights” debate, we decided, was really a red hearing. Rather, we both agreed the real issue is how we will achieve or at least show steady forward progress toward UHC.

When Soji, Mickey Chopra and their colleagues from the World Bank, Nanda and his colleagues from USAID, and I met, it was clear there was a need to critically examine these issues not only from a policy perspective but equally importantly from the perspective of countries actually implementing UHC. Each paper in this series addresses some fundamental problem in the achievement of UHC. The problem is best encapsulated in the first paper, “The Roads to Universal Health Coverage: Manifest Destiny or Sisyphean Pursuit” by Olusoji Adeyi, Mickey Chopra, and Allyala Nandakumar when they state:

“The reality for millions of people across the world is that UHC remains an elusive goal despite global proclamations and resolutions, and despite statements of intention by policy makers at the country level”* [**1**].*

The mismatch between expectations and realities is at the core of the papers in this volume and how to resolve what can be, and what certainly will be, a volatile mismanagement of expectations. All the tools we have at our disposal as economists, financial experts, governance specialists, and health experts must be brought to bear to set the table for feasible and sustainable solutions.

Will we meet the UN and SDG goals of UHC by 2030? In my view, not likely. That is, not unless we cynically reinterpret the term “universal.” But no one wants to reduce our solutions to semantics. And to do so could be explosive; for while I disagree with placing health care as a fundamental right in the constitutions of countries across the world, the fact that it is a movement of considerable popularity means solutions must be forthcoming and results will be critical –no matter the legal definitions.

One key debate and theme that emerges from the papers in this volume, of which I am proud for the diversity of viewpoints on, is the extent to which the public and private sectors, or the mix thereof, can put a country on the path to UHC. Some papers clearly emphasize their confidence in a more paternalistic view of “health care for all.” Viroj Tangcharoensathien, Walaiporn Patcharanarumol, Anond Kulthanmanusorn, and Ariel Pablos-Mendez’s paper *“Paths towards universal health coverage: Beyond political commitments”* makes the case for greater state control of the provision of services and cite the example of Thailand with some success [2]. However, other papers such as *“Health financing reforms for universal health coverage in five emerging economies”* by Chris Atim (Ghana), Indu Bhushan (India), Mark Blecher, Jonatan Daven (South Africa), Ramana Gandham (Kenya), Vikram Rajan (Indonesia); and Olusoji Adeyi; show the government’s share in health spending on UHC goals needs to be increased as a share of GDP when growth rates are accelerating. However, as they point out, this does not necessarily imply government-run health care is necessary to achieve UHC goals [3]. Rather a proper balance between the private and public provision on health services options is the key – in most cases governments need to keep pace with the innovative and demand-driven private sector services’ provision.

The case of India and its focus on the private provision of services in combination with a smaller government sector illustrates the likely future of UHC success. Indu Bhushan’s paper in the five-country comparison has a very revealing discussion on the success that India is having of late where they have handed over whole portions of health services provision to the private sector. In these less paternalistic models, while they show greater success of advancement toward UHC, they struggle with the proper regulatory oversight and the sustainable policy mix required by their governments to ensure steady progress.

In some cases, it is not an ideological issue – often the debate needs to be first formed in the context of reliable data. The need for simply better evidence and better data are highlighted quite well (and informatively) by Chris Atim, Augustina Koduah, and Soonman Kwon in their article *“How and why do countries make UHC policies? Interplay of country and global factors*”. Here the problems and the various solutions are discussed in detail [4].

Basic distributional and fairness issues, so critical to the core of the debate and often overlooked, are elegantly laid out by Ajay Tandon and K. Srinath Reddy in their paper *“Redistribution and the Health Financing Transition”* [5]. The critical digital solutions that are absolutely necessary as technical inputs to long-run solutions are spelled out and discussed in David Wilson’s, Aziz Sheikh’s, Marelize Görgens’, and Katherine Ward’s paper *“Technology and universal health coverage: Examining the role of digital health”* [6].

Finally, a broad sweeping political economy review of the longer-term issues involved in achieving UHC is aptly provided by Alexander S. Preker, Daniel Cotlear, Soonman Kwon, Rifat Atun, and Carlos Avila in *“Universal health care in middle-income countries: Lessons from four countries”*. They point out that perhaps the most necessary step toward a country’s ability to achieve UHC is a political-economy one, and not simply the calculus of fiscal policy [7]
